# School Green Space and Its Impact on Academic Performance: A Systematic Literature Review

**DOI:** 10.3390/ijerph16030429

**Published:** 2019-02-01

**Authors:** Matthew H. E. M. Browning, Alessandro Rigolon

**Affiliations:** Department of Recreation, Sport and Tourism, University of Illinois at Urbana-Champaign, Champaign, IL 61802, USA; rigolon@illinois.edu

**Keywords:** green space, nature, academic performance, academic achievement, education, schools, test scores

## Abstract

*Background*: Scholars and policymakers have criticized public education in developed countries for perpetuating health and income disparities. Several studies have examined the ties between green space and academic performance, hypothesizing that green space can foster performance, and, over time, help reduce such disparities. Although numerous reviews have analyzed the link between nature and child health, none have focused on academic achievement. *Methods*: We identified 13 peer-reviewed articles that examined associations between academic outcomes, types of green spaces, and distances in which green spaces were measured around schools. *Results*: Of the 122 findings reported in the 13 articles, 64% were non-significant, 8% were significant and negative, and 28% were significant and positive. Positive findings were limited to greenness, tree cover, and green land cover at distances up to 2000 m around schools. End-of-semester grades and college preparatory exams showed greater shares of positive associations than math or reading test scores. Most findings regarding writing test scores were non-significant, and moderation effects of socioeconomic status, gender, and urbanization showed mixed results. *Conclusions*: The extant literature on green space and academic performance is small, shows mixed results, and mostly includes articles using observational, school-level research designs. Regardless, there is sufficient evidence to warrant further research on this topic, including effect moderation and mechanistic pathways.

## 1. Introduction

Public schools are tasked with preparing students to fulfill their potential, lead satisfying and productive lives, and be ready for college, workforce, and civic life [1,2]. Academic performance—including test scores and grades—is an important predictor of success and wellbeing in adulthood. More specifically, students who demonstrate better performance at school or in college are more likely to earn higher salaries [3,4], engage more as active citizens and vote more in political elections [5,6,7,8], report higher life satisfaction and happiness [9], and participate in less illicit behavior than those with lower scores. Yet, in the United States and other developed countries, schools serving predominantly urban, low-income populations are struggling [10]. Sixth graders (generally aged 11 to 12) in the richest school districts are four grade levels ahead of children in the poorest districts; there are large gaps between non-Hispanic White children and their Black and Hispanic classmates; and the gaps are largest in places with large economic disparities [11]. Children who attend urban schools in low-income neighborhoods have shown the lowest academic achievement in the country for decades [10,12]. In the absence of effective solutions to poverty and discrimination, policymakers need to identify low-cost interventions that help disadvantaged urban children reach their potential.

A growing body of literature points to the tantalizing possibility that green space around schools could boost academic achievement. Recent experimental work in school settings echoes a large body of research on the restorative effects of contact with nature on underlying factors required for success in school, including attentional capacity and low stress levels [13,14]. Views of greenery from classroom windows improve concentration and reduce both self-reported stress and heart rate, whereas classrooms without green views do not [15]. Teaching outdoors or in natural or agricultural areas can also aid learning comprehension and retention [16,17,18,19]. And learning in relatively green classrooms, in school gardens, and in natural contexts has been associated with high levels of student interest [20,21,22]. Although there have been numerous reviews of studies of green space benefits for childhood health and wellbeing [23] and outdoor education [24], no systematic review has focused on the relationship between school green space and academic outcomes. Reviewing this literature would inform school greening interventions to boost academic achievement.

In this systematic review, we examine the available evidence on the connections between green space around schools and student’s performance. We focus on studies that have explicitly looked at young adult and child academic outcomes, including standardized test scores, end-of-semester grades, and college preparatory exams. We choose these measures because they provide more consistent metrics for comparing academic achievement across schools than other metrics [25,26]. For instance, we did not to include assessments of individual lessons or curricula delivered in outdoor settings, which are not comparable across schools and therefore represent a related but separated body of literature [16,17,18,19]. 

We focus on green space within and around school campuses rather than near students’ home for three reasons. First, school administrators have more control over these areas, as they can make decisions about school ground greening or work with the surrounding communities to implement other greening initiatives [27]. Second, the greening of school grounds requires relatively little financial investment [28]. Third, increasingly common school choice programs—which involve the opportunity for families to choose schools within a school district, regardless of place of residence [29,30]—make it more effective to implement greening programs in and around schools than in the spatially scattered neighborhoods where students reside. 

The overall objective of this review is to identify, critically appraise, and synthesize the findings of studies examining the relationship between green space near schools and academic performance. Specifically, we ask:

(1) What is the strength of evidence tying school green space to academic performance?
(2) How do study findings differ by the measure of academic performance considered?
(3) How do study findings on this topic differ by the measure of green space considered?
(4) What effect do confounding and moderating variables have on green space and academic performance associations?

## 2. Materials and Methods 

**Figure 1:**
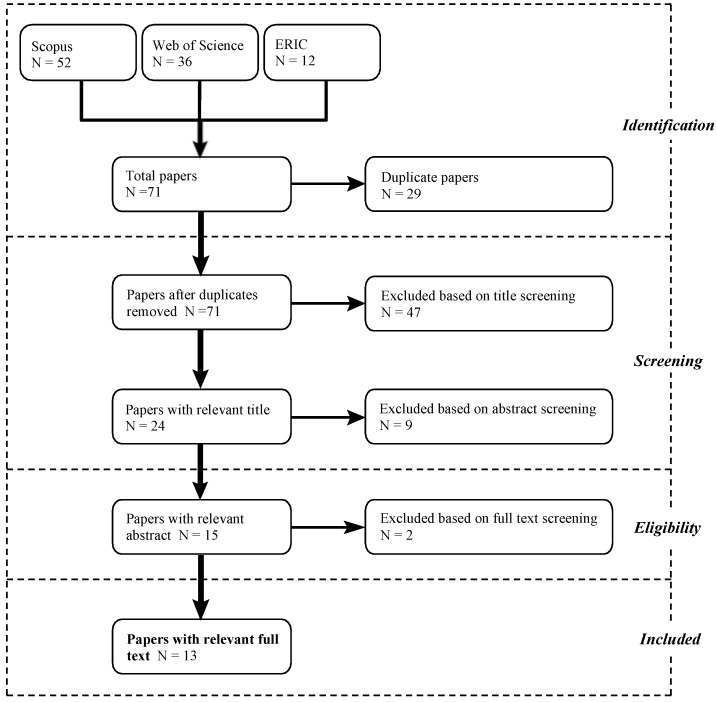
PRISMA flow diagram depicting results of search, screening and selection processes.

For this review, we followed the guidelines outlined in the Preferred Reporting Items for Systematic Reviews and Meta-Analyses (PRISMA), which provides rigorous standards for performing and reporting systematic reviews [31]. This protocol describes four steps in the selection of empirical articles: identification; screening, which involves reading titles and abstracts; eligibility, involving reading the full texts; and inclusion (Figure 1). 

First, we identified potential papers through Scopus, Web of Science, and Education Resources Information Center (ERIC) searches on 12 December 2018, using keywords related to green space and academic performance (see Appendix A, Table A1). We chose these databases because they included peer-reviewed journals in disciplines that have studied how the provision of green space relates to academic achievement, including education, urban studies and planning, geography, sociology, and environmental studies [32]. We also reviewed the reference lists of all eligible articles and screened additional potentially relevant studies with the same selection process as the keyword search; however, this did not result in additional eligible articles.

Second, both authors independently reviewed titles, abstracts, and full texts to make decisions about article eligibility. For inclusion in this review, studies had to meet four criteria:

(1) Report at least one objective measure of green space within or around school campuses. In this paper, we use the phrase “green space” to describe areas of vegetation, such as forests, street trees and parks, and gardens [33]. We define “within or around school campus” as the area describing students’ experience of nature at school. This includes not only the school property but also the 25 m buffer around the property. This larger area represents the viewshed in which students may visually or physically access green space during the school day [28].
(2) Report at least one objective measure of students’ achievement. We focus on indicators of success strongly tied to long-term measures of educational achievement and career success, including standardized tests scores [20,21,22], grades [34,35], and college preparatory exams [35].
(3) Perform any type of inferential statistical test (i.e., correlations, regressions, t-tests) to examine the relationship between green space around schools and academic performance.
(4) Present original research findings in peer-reviewed journals written in English.

One author (M.H.E.M.B.) extracted key data from each eligible study into a Microsoft Excel spreadsheet, and the other author (A.R.) validated these data. Data elements included: authors, journals, publication year, location (e.g., continent, country or city), sample characteristics (e.g., grade level, number of individuals or schools), study design (e.g., experimental or observational), unit of analysis (e.g., individual or school), academic achievement outcome measured (e.g., reading test scores or end-of-semester grades), green space data resolution (e.g., 30 m or 250 m), green space type (e.g., tree cover or grass cover), season in which green space data were collected (e.g., spring or summer), distance from school in which green space was measured (e.g., schoolyard only or 1000 m radial buffer centered on school address), confounding variables (e.g., student-teacher ratio or gender), moderation effect tests (i.e., interaction terms or sensitivity analyses), statistical approaches (e.g., linear regression models or paired sample *t*-tests), and significance and direction of findings. 

When articles reported multiple findings—for instance, associations between trees and math versus reading standardized test scores—we extracted data for each analysis. We did not conduct statistical meta-analyses of study findings due to inconsistencies in study design, exposure measurements, comparison techniques, statistical analysis, and reporting methods. Rather, we report frequencies and counts of study characteristics including the academic outcome measured (e.g., math standardized test scores), type of green space (e.g., trees), and distance from school at which authors measured green space (e.g., up to 250 m).

To address Question 1, we report how many studies found significant (positive and negative) and non-significant associations between green space and academic achievement as well as report an evaluation of the methodological biases present in the reviewed studies. We adapted an evaluation bias instrument from the International Agency for Research on Cancer framework, which is used for evaluating mechanistic evidence [36] and has been used in other reviews on human benefits of green space [37,38,39]. We focused on four categories for evaluating bias relevant to this review: study design, confounding, statistics, and exposure assessment (Table 1). 

We selected the items for each category based on weaknesses identified in prior systematic literature reviews on green space and health [39] and green space proximity and accessibility [32]. Because one author (M.H.E.M.B.) co-authored two papers in this review, the other author (A.R.) first conducted bias evaluations, and then the first author (M.H.E.M.B.) validated the results. For each of the four criteria, we assigned up to four points to each article. Subsequently, we converted the sum of these points to a percentage of the maximum score. The maximum score was equal to 16 for most studies and 12 for two studies not using large geospatial datasets (e.g., NDVI) to measure green space [48,49]. We then assigned each paper a summative quality measure based on established cut-off values for the percentages of points assigned [37,38,50]. The five levels of quality were excellent (score ≥ 81%), good (between 61% and 80%), fair (between 41% and 60%), poor (between 21% and 40%) and very poor (≤ 20%).

To address Questions 2 and 3, we counted significant versus non-significant findings by educational outcome and measure of green space. Had we counted all models in all studies, we would have undervalued the contribution of studies that reported fewer models. To weight each studies’ findings equally, we summarized each possible analysis into a single entry. For each entry, we identified one of five academic performance outcomes (math test scores, reading test scores, writing test scores, grades, or college preparatory exam scores); one of seven distances from school in which green space was measured (window view; schoolyard; radial buffers or polygons up to 250 m, 500 m, 1000 m, or 2000 m in size centered on the school; or radial buffers or polygons 3000 m or greater that included the school), and one of six green space types (agriculture, grass cover, green land cover, greenness, shrub cover, or tree cover). In total, each study could contribute only once to each of 210 categories (5 outcomes × 7 distances × 6 types). However, because Markevych and colleagues [51] reported findings from two separate populations, their article could contribute twice to each category—one entry for each sample.

In counts by outcome and green space measure, we compressed articles with multiple findings per category into a single entry. For each category, if at least 50% of findings were significant, we coded findings for that category as significant. For example, Beere and King [47] reported one negative and one null association between reading and green land cover at 3000 m or greater distances, which differed based on whether the authors considered or not the combination of public and private green space; for this article, we coded the link between green land cover at 3000 m or greater distances and reading as negative and significant. If all findings were significant for a given category, we coded that article for that category as having a significant finding in the direction of the greatest number of findings. For example, Wu et al. [52] found two positive associations and one negative association between math and greenness at 1000 m, so we assigned a significant positive finding to this article for this category.

Outcomes and measures of some articles did not fit precisely into categories. For example, Tallis and colleagues [53] reported results from an index of math, reading, and writing test scores. This article received separate math, reading, and writing finding for each green space measure examined; elsewhere we coded the omnibus outcome, “language art,” as reading unless accompanying citations clearly indicated writing was also tested; in this case, tests were coded for both reading and writing outcomes. Tallis and colleagues [53] also reported findings at 10 m, 50 m, and 100 m radial buffers centered on the coordinates of the school; we counted these metrics as green space measures on the schoolyard. Another example of an imperfect category match is from Kweon et al. [54], who used a green space measure that included both grass and shrub cover. We assumed that associations with this measure represented grass and were assigned to this category exclusively because, in another study in Minnesota’s Twin Cities, high-resolution satellite imagery suggests grass is the dominant green space type in schoolyards and school attendance zones [55].

**Figure 2:**
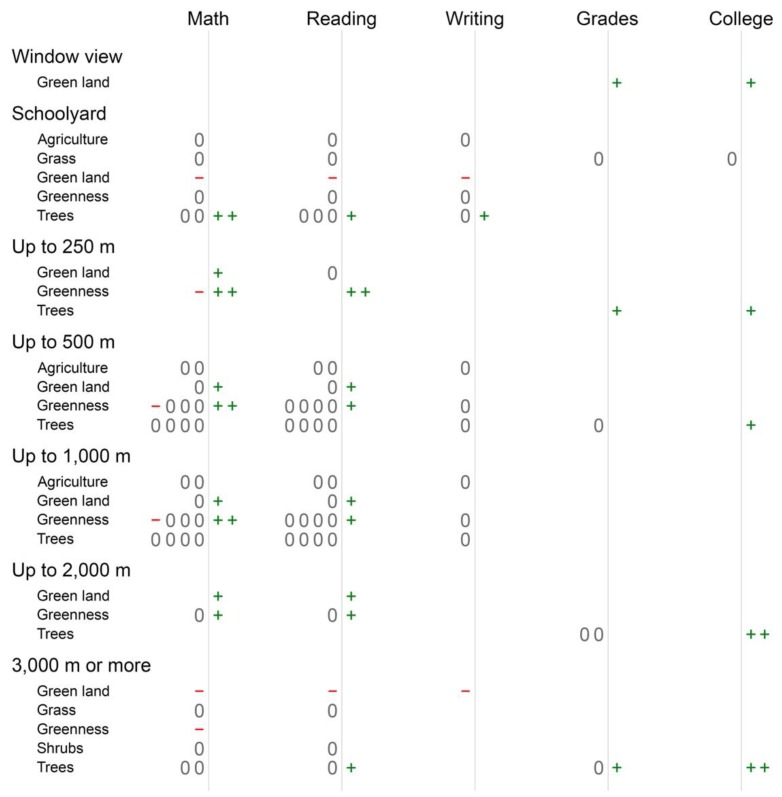
Counts of findings by outcome and green space measure with symbols representing the following associations between academic performance and green space: + = statistically significant positive association, 0 = non-statistically significant association, and - = statistically significant negative association. Metric distances (e.g., “Up to 250 m”) represent the radius of circular buffers or diameter of polygons centered on a school. Statistical significance requires *p* < 0.05 for 50% or more of analyses summarized in articles that included a measure or proxy for socioeconomic status (i.e., the percentage of students eligible free-or-reduced lunch) because SES often predicts green space cover and vice-versa [57,70,71]. As such, bivariate correlation coefficients were not included in this summary.

To develop answers to Questions 2 and 3 (reported in Figure 2 below), we only considered statistical analyses in the selected articles (*n* = 13) that controlled for socioeconomic status (SES). We did so because there is a strong link between academic performance and SES [10,56]. Also, poorer neighborhoods have less green space cover, fewer acres of parks, and parks with lower quality than wealthier areas [32,57]. Similarly, schools in poorer communities have less green space than those in wealthier areas [28]. Thus, if we included analyses that did not adjust for SES, such as bivariate correlations, their results on the connections between green space and academic performance might be spurious due to the unaccounted effects of green space differences across the SES gradient.

Finally, to address Question 4, we describe the study results on the effect of possible confounding and moderating factors. Specifically, we report confounders used in studies and we report findings from models with interaction terms (i.e., SES * green space) as well as sensitivity analyses with split samples (i.e., low-SES vs. high-SES schools). These estimated whether effects of green space on achievement differed by certain school or student characteristics.

## 3. Results

### 3.1. Article Selection

The initial database search produced 71 results, but the majority were not relevant for this review. Only 13 full-text articles met inclusion criteria, suggesting that the literature on school green space and academic performance is still at its early stages. Articles contained one [49] to 176 [51] inferential statistical analyses that also met inclusion criteria. In total, 574 analyses were included in this review.

### 3.2. Descrition of Articles

Articles were largely published in recent years in interdisciplinary journals. Nearly all (10 out of 13) of the studies included in the review had been published since 2014. Journals primarily spanned the social and environmental sciences (i.e., *Landscape & Urban Planning*, *n* = 3; *Urban Forestry & Urban Greening*, *n* = 2; *Environment and Behavior*, *n* = 1; *Environmental Pollution*, *n* = 1) but also included a 2018 special issue in *Frontiers of Psychology* (*n* = 2), *PLoS ONE* (*n* = 2) and a geography journal (*New Zealand Geographer*, *n* = 1). Over one-fourth (4 of 13) of articles were open-access, providing manuscripts to interested readers (e.g., school administrators) without a subscription to academic publishers. While articles in education journals (i.e., *Environmental Education Research*) were identified in the selection process of this review, none qualified for inclusion. In addition, all 13 articles focused on schools and students in Global North countries, and predominantly (10 out of 13) in the United States.

### 3.3. Study Design and Quality

Table 2 shows study and population characteristics organized by study design. Sample sizes for school-level studies ranged from 101 [48] to 6333 [52]. The two individual-level studies included 567 students [49] and 2429 students [51]. Studies commonly focused on urban school districts, including Munich, Germany [51] and several cities in the United States like Chicago [28,58], Washington D.C. [54], the Twin Cities [55], and Boston [52]. The Munich and Boston studies also reported findings for other areas of Germany and the state of Massachusetts, respectively. Most studies (10 of 13) reported findings on elementary school children (3rd or 4th grade, typically 8–10 years old), and three studies included findings from middle school (7th to 8th grade, typically 12–14 years old) [51,54,59]. Five also included findings at the high school level (9th to 12th grade, typically 14–18 years old) [48,51,59,60]. One included findings at the college level (typically 18–21 years old) [49].

The majority of studies were observational and measured standardized test scores. Many studies tested for dose-response curves by measuring green space at varying distances from the school [47,51,52,53,58,59,60]. In the only experimental study included in this review, the authors sought to identify causal mechanisms related to exposure to green space by examining whether students with window views of trees and green space during a semester-long writing course had better scores than students in classrooms without windows [49]. 

**Figure A1:**
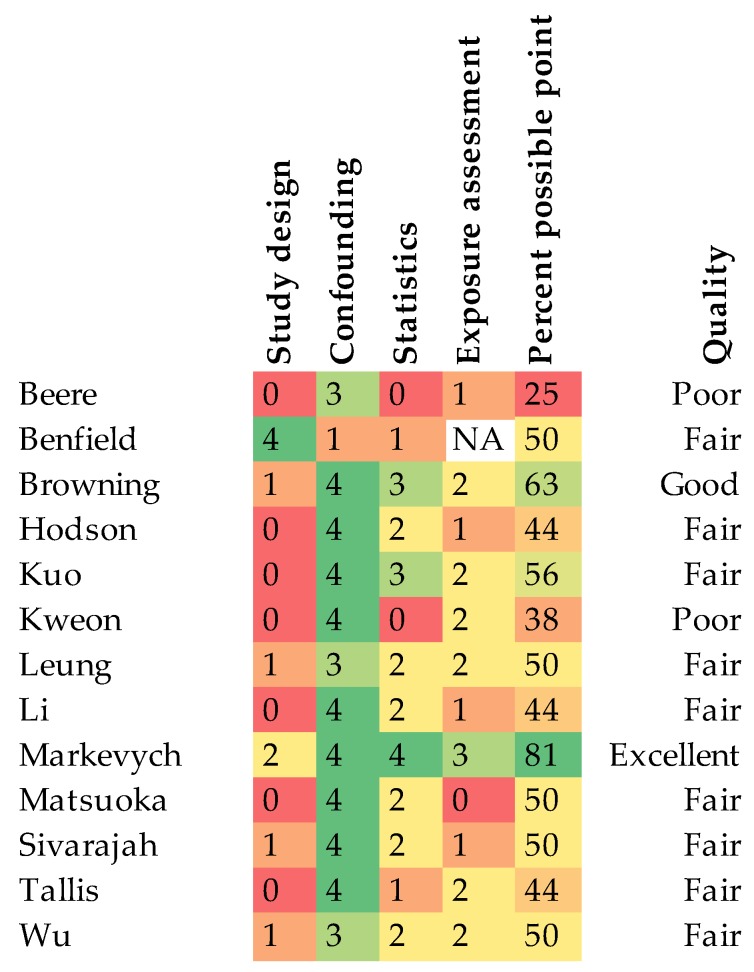
Points assigned to bias categories for articles in the review.

Our analysis of methodological biases showed that the majority of the included studies (*n* = 9) were of fair quality [28,48,49,52,53,55,59,60,61]. Only two were of good [58] or excellent quality [51], and two more were of poor quality [47,54]. Common potential biases were single-year and single-season measures of green space, inadequate control for spatial autocorrelation and collinearity between variables in regression models, and coarse green space resolution measures. Scores for each bias category of each article are provided in Figure A1 in the Appendix A.

### 3.4. Differing Associations by Outcome and Green Space Measure

Figure 2 reports counts of the number and direction of statistically significant and non-significant findings by category. Categories represent the combination of one academic achievement outcome, one type of green space, and one distance in which green space was measured. Each article could contribute no more than one finding (positive, negative, or null) per category (see Section 2).

Article findings represented 122 entries in 66 analysis categories. Because there were 210 categories (five academic outcomes × seven distances in which green space was measured × six green space types), only 31% (66 of 210) of the possible associations were studied in the reviewed papers.

Most findings were non-significant. Of 122 entries, 78 (64%) were null, another 34 (28%) were positive [28,48,52,54,55,59,60,61], and ten (8%) were negative [47,58]. Support for a beneficial impact of green space on academic outcomes would have been observed in categories with large numbers of positive findings; however, we observed no more than two positive findings for any given category (see Figure 2 and Table A2 in Appendix A). On the other hand, three categories had two positive findings and zero non-significant or negative findings. These categories were trees and college preparatory exams at 2000 m and 3000 m or greater distances, and greenness and reading test scores at 250 m distances. Other categories showed two positive association counts but also non-significant or negative findings. These included math, reading, and college outcomes for tree and greenness measures at all distances, except window views.

Findings varied widely by academic achievement outcome. The most promising findings for the influence of green space on achievement were for college preparatory exams results; of eight total findings, seven (88%) showed beneficial associations between green space and achievement [48,60]. Moreover, we found exclusively positive associations between college exams and green land cover or tree cover at all distances [28,54,55,60,61]. End-of-semester grades also showed exclusively positive associations for green window views and trees in up to 250 m distances away from schools [48,60]. However, both college preparatory exams and end-of-semester grades were studied infrequently; in other words, the total counts of articles were low compared to other academic outcomes. End-of-semester grades, math, and reading tests scores showed lower ratios of positive findings when examining any green space measurement: three of eight (38%), 13 of 49 (27%), and 10 of 45 (22%), respectively, were positive. Writing test scores showed the lowest share of positive findings: nine associations were null, two were negative, and only one was positive (8%) [47,53,61].

We observed positive associations for three types of green space. Nine of 20 findings (45%) for green land cover were positive. Greenness and tree cover showed similar percentages of positive findings: 12 of 37 (32%) [52,59] and 13 of 44 (30%) [28,54,55,60,61], respectively. We did not find any positive findings for agriculture, grass, or shrub cover [48,51,53,54,55].

Examination of green space distances showed patterns for window views (positive), 2000 m distance (positive), and 3000 m or greater distances (negative). Window views showed particularly promising support for beneficial associations between green space and academic achievement; all findings were positive, but only two findings were reported [48]. Associations at distances up to 2000 m were also promising: six of ten (60%) were positive and none were negative [59,60]. In contrast, only three of 12 findings (25%) at 3000 m or greater distances were positive [55,60], whereas four of 12 (33%) were negative [47,58].

Finally, several categories showed exclusively negative associations. These included green land cover and math, reading, and writing outcomes for schoolyards and 3000 m or greater distances [47,58]. Greenness also showed exclusively negative associations at the 3000 m or greater distance [58].

### 3.5. Differing Associations by Confounders and Moderators

We identified 30 different confounding variables and moderating factors in the 13 included studies. The most common were socioeconomic status (SES), race and ethnicity, gender, student-teacher ratio, student attendance, and urbanization (i.e., urban and rural). Less common variables included school resources (e.g., classroom size, expenditures, teacher retention rates), environmental characteristics besides green space (e.g., public vs. private land, water bodies), and support for students outside of typical classroom instruction (e.g., individualized learning plans, parental involvement). The six papers that included moderation models examined the following relationships: SES * green space [28,51,52,59,61] urbanization * green space [51,53], and gender * green space [51,52]. 

Four of five studies found significant moderation effects for SES [28,52,59,61]; however, the direction of these effects varied. Two studies provided evidence that students in low-SES schools benefited less from green space in terms of academic achievement [52,59]. A third study showed the opposite [61], and a fourth study reported no clear pattern in the results [28]. The third study [61] examined moderation effects from a “learning opportunity index” that included a broad range of SES variables (e.g., median income, parental education levels, percentage of families receiving social assistance), while the first two studies [52,59] used a single indicator representing the percentage of students eligible for free or reduced lunch. As such, the differences in findings may be partially attributable to variations in SES measurements. Given that SES is a multidimensional attribute that extends beyond a families’ reported salary [72], the evidence for a greater benefit for schools with poorer student bodies from Sivarajah and colleagues [61] may be more robust than that of studies using free or reduced lunch as the only SES variable [52,59]. On the other hand, the single study that did not find significant moderation effects may provide even more reliable evidence on whether a moderation effect exists since it used individual-level academic performance and SES data [51].

Findings on the moderating effects of SES might also vary by geographical context. The two studies showing that students in low-SES schools benefit less from green space were based in the state of Massachusetts, which includes a broad range of rural, suburban, and rural areas [52,59]. And the study showing that students in low-SES schools benefit more from green space was based in Toronto, Canada [61], a much denser urban metropolis than most of the areas in Massachusetts. Green space may matter more for the academic achievement of low-SES students in urban areas—where green space tends to be scarcer—than for the achievement of low-SES students in suburban and rural areas—where green space is more available. On the other hand, findings for effect modification across urbanization levels are unclear (see below). Collectively, these studies do not provide clear evidence for whether SES moderation is present and how it impacts the relationship between school green space and academic performance.

Examinations of the moderating effects of gender and urbanization were uncommon and largely non-significant. One study across California public schools found urban schools benefit more than rural schools [53], which might confirm the above hypothesis on the importance of location when considering SES as a moderator. A second study across two areas of Germany found no moderation effects of urbanization [51]. Two of three studies that examined gender also found no moderation effects [51,59]. A third study showed schools with more females benefited more from green space than schools with fewer females [52]. Ultimately, this limited number of studies does not clearly identify demographic groups or geographic regions that benefit the most from green space near schools.

## 4. Discussion

Academic performance is an important predictor of health, wellbeing, civic engagement, and socioeconomic status in adulthood [3,4,5,6,7,8,9]. Public schools serving disadvantaged students—such as low-income and racial and ethnic minority students—will likely perpetuate existing and growing health and income disparities in the United States and other developed countries [73,74] if they do not provide adequate educational opportunities for their students. Scholars and policymakers are increasingly interested in low-cost academic performance interventions, including school green space [23].

To evaluate green space’s potential as an academic intervention, we conducted a systematic review of studies tying green space within and around school campuses to academic performance. We found that the extant literature (*n* = 13 articles) provides only weak evidence for this potential intervention. The vast majority of analyses showed no statistically significant relationship between these variables and, although 28% of findings showed a significant and positive relationship, another 8% of findings showed a significant and negative relationship between school green space and academic performance. 

The limited number of studies we identified signals that the literature on school green space and academic performance is in its infancy. This is, in itself, a finding of this review. The paucity of studies on school green space and achievement contrasts with the burgeoning body of literature on the beneficial impacts of green spaces on human health; one recent review identified 143 articles on green space and human health [75]. On the other hand, there are also precedents of other systematic reviews that found small numbers of relevant papers when reviewing a focused body of research, such as is the case with the current review. For example, one review found five articles on green space and childhood atopy [76] while another found 11 [77]. Other reviews included eight articles on young adults’ experiences in remote green spaces [78] and 18 studies on the psychosocial benefits of green space for people with dementia, brain injury, or stroke [79]. Yet another review included 13 articles on the impacts of outdoor education programs on students’ health, wellbeing, and learning [14].

### 4.1. Overview of Study Limitations

Twelve of 13 studies employed an observational design, which cannot directly support the argument for a causal relationship between green space and academic performance. Because this body of literature is in its infancy, it is appropriate and expected for scholars to focus on basic research and theory building rather than cause-and-effect relationships [80]. Yet this body of observational work might borrow from epidemiology and provide conceptual frameworks, or directed acyclic graphs (DAGs), that represent an overview of causal mechanism under scrutiny [51,81,82].

In this review, we identified one experimental study that suggested a cause-and-effect relationship between academic achievement and semester-long exposure to green space views from windows [49]. This single study alone supports the promise of a causal relationship between these constructs. Interestingly, the observational studies in this review generally showed green space in schoolyards had no relationship with writing test scores [47,53,61]. This discrepancy highlights the need for more research on the effects of schoolyard green space on writing outcomes.

That nine of 12 studies were only of fair quality suggests this body of literature is not yet methodologically robust. However, other reviews of mental health and cognitive benefits derived from green space also find a preponderance of fair quality evaluations among the included studies [38,50]. Collectively, the current review supports others in its conclusion that we need more evidence for the beneficial effects of green space before we can confidently draw conclusions.

### 4.2. Patterns Linking Green Space to Academic Performance

Keeping the above limitations into account, we identified some patterns in how academic outcomes were related to green space measurements. Trees near schools and green window views showed greater numbers of exclusively positive findings with academic performance than other types of green space. Also, college preparatory exams and end-of-semester grades showed greater numbers of exclusively positive findings with green space than other measures of academic performance.

Greater numbers of null or negative findings were found for the following: writing test scores; agricultural, grass, and shrub covers; and distances in which green space was measured far away from schools (i.e., 3000 m or greater distances).

Higher shares of null and negative findings for writing scores could be explained by writing causing less anxiety than other academic subjects or specific tests. For example, mathematics as a subject [83], final exams that determine end-of-semester grades [84], and college-preparatory exams [85] cause tension and fear in many students. These feelings manifest themselves in test anxiety that disrupts concentration, and ultimately, performance [86]. If writing causes less test anxiety, students would be better able to concentrate. Subsequently, attentional restoration derived from green space would be less pronounced.

Studies that measured green space in greater distances might have found null or negative associations because these sizes represent neighborhood rather than school green space. Importantly, these measures might not represent the green space that students are exposed to on a daily basis, especially if they do not live in the school’s neighborhood, as in the case of schools of choice. Even if students live in the neighborhoods captured at larger distances, one study found the association between neighborhood green space and academic performance is only marginally statistically significant (*p* < 0.10) when school green space is accounted for [28]. Thus, neighborhood green space may have little effect on academic performance, because measuring green space at larger distances dilutes any beneficial impact of green space near schools.

Null and negative associations between academic performance and agriculture, grass, and shrubs could be explained by trees providing more benefits than other vegetation. Trees filter out air pollutants better than other types of vegetation [87], and air pollution is negatively linked to academic performance [88,89]. Indeed, correlational evidence suggests the provision of clean air partially explains green space’s impact on its attentional benefits [90]. Trees also help lower air temperature, which, in hot climates, extends the number of days that vulnerable populations, like children, can comfortably spend time outdoors [91]. Last, many students find trees aesthetically pleasing and, therefore, prefer schoolyards with trees to schoolyards without trees [92]. Because landscape preferences are linked to psychological restoration, the aesthetics of trees may also explain their enhanced benefit over other vegetation types [93].

### 4.3. Future Research

We identified several areas for future study of green space and academic performance. These are motivated by the need to overcome potential biases in research methods, to further explain the mixed findings of this review by examining mechanisms, and to study this important topic in currently understudied geographic areas such as Global South countries. 

First, to address methodological biases, data obtained at the individual-level would avoid ecology fallacy, which emerges from aggregated (or ecological) data that cannot be used to make assumptions about associations at individual levels [94]. Measuring green space during multiple seasons and for several years would control for climatic and annual fluctuations affecting measurement [43,44,45]. Limiting collinearity issues between confounders, addressing spatial dependence between spatially-arranged objects (i.e., schools), and controlling for time dependence in multi-year datasets (i.e., repeated measures of academic achievement) would strengthen the reliability of model coefficients and reduce non-random error [42,95]. Finally, finer-scale resolutions of green space (i.e., at least 30 m or less) would prevent under- or over-estimating green space across urban-rural gradients [46].

Second, to explain the mixed findings of the current body of work, future research should focus on the mechanisms by which green space may (or may not) boost academic performance using robust study designs that adequately account for confounding and moderating factors. Markevych and colleagues proposed a complex conceptual framework to help understand such mechanisms [51]. They showed how green space relates to children’s time in green space, family socio-demographic characteristics, mental health, and ultimately to academic performance. Other scholars (i.e., Leung and colleagues [59]) provided a written description of what outcomes emerge from time spent in green space. Specifically, green space can help reduce stress and restore mental fatigue [96,97], increase concentration and attentiveness [98], support cognitive development [99], increase self-discipline [100], and boost classroom engagement [101]. In the long-term, scholars argue, these benefits would help students absorb academic content and perform better on tests [102]. Because the only experimental study identified in this review found strong evidence linking green space views from windows—not time spent outdoors in green space—to achievement, the pathway between green space presence and cognitive functioning is particularly promising and deserves further investigation [49]. 

**Figure 3:**
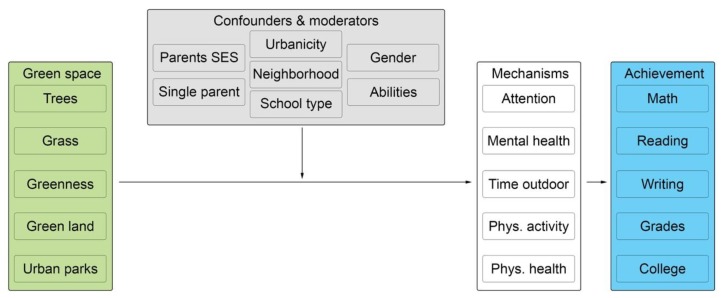
Conceptual framework adapted from the model proposed by Markevych and colleagues linking green space near schools to academic performance [51].

To synthesize the mechanisms that link green space to academic achievement, we provide a conceptual framework that identifies potentially relevant variables for future research (see Figure 3). We adapted this framework from the one proposed by Markevych and colleagues [51] and incorporated pathways identified in another study in this review [59]. Different types of green space could influence different academic outcomes through one of five mechanisms: attention restoration, better mental health, more time outdoors, more physical activity, and better physical health. A number of confounding and moderating factors may affect the relationships between green space and these mechanisms. Such factors include, among others, parental characteristics (e.g., SES including income and education, and single-parent status), school and environmental factors (e.g., magnet vs. neighborhood vs. charter school, urban vs. rural settings, and neighborhood characteristics such as crime, traffic, and air quality), and individual factors (e.g., gender, cognitive abilities, and eyesight). 

Among the possible confounders and moderators, poor eyesight is a major barrier to academic performance [103,104], and viewing green space activates at least three of the mechanistic pathways by which academic performance may be achieved: attention, mental health, and physical health [105,106]. Exposure to green space—particularly around schools—has also been linked to lower rates of vision degeneration [90], such that greater school green space might prevent the need for eyeglasses and improve academic performance. Air quality represents another important but understudied environmental variable. In one study, less air pollution around greener schools explained up to 65% of the association between school green space and improved attention [107]. Although greener schools have higher attendance rates [108], which is another predictor of academic success [109], green space filtering out air pollutants might explain these positive effects [108]. 

Third, in our review, we did not find any studies outside of Europe and North America, suggesting that scholars might not have studied the green space-academic achievement link in Global South contexts or that studies of these locations might be published in languages other than English. Thus, we recommend future research on green space and academic performance study populations and schools outside of the Global North. The dearth of studies in Global South countries might signal the current body of research has missed additional possible factors confounding the relationship between green space and achievement, since cultural differences can modify the relationship between green spaces and perceived benefits [110]. That most studies we identified focused on the United States is not surprising, as school administrators and policies in the United States place particular emphasis on measures of academic achievement, such as test scores [111]. But the lack of studies on green space and academic achievement in Global South countries is indeed unexpected and relevant to future researchers, as studies of green space access and use in these contexts has been extensive [32]. Ultimately, consideration of a range of pathways, confounders, and mechanisms will help researchers develop theory on this intriguing topic.

## 5. Conclusions

In conclusion, the extant body of literature examining school green space and academic performance is small, shows mixed results, and is dominated by articles of only moderate quality presenting multiple methodological limitations, specifically a predominance of observational, school-level study designs. Despite these limitations, we found sufficient evidence to warrant further research on this topic. Consideration of confounding, moderation, and mechanistic pathways should be the focus of future investigations. At this time, tree cover near schools, green window views, college preparatory exams, and end-of-semester grades are the most promising indicators of a beneficial link between school green space and academic performance.

## Figures and Tables

**Table 1:** Methodological biases in sampled papers.

Bias Category	Biases Identified
Study design	Randomized control trial rather than observational data (4 pts.) For observational studies, multiple years of data for outcome variable (1 pt.) For observational studies, individual-level data for outcome variable (1 pt.)
Confounding	Adequate control for confounding variables, specifically socioeconomic status (SES) (2 pt.) Rationale for selection and inclusion of control variables (little or no rationale = 0 pt., empirical or theoretical rationale = 1 pt., both empirical and theoretical rationale = 2 pt.)
Statistics	Used appropriate statistical analyses for given dataset(s) and research question(s), such as a detailed description of the statistical technique used, explanation why this technique was chosen, and discussion of caveats regarding the conclusions drawn from analyses using this technique [40] (1 pt.) Performed sensitivity test(s), for instance, differential effects by urbanization, gender, SES, or distances in which green space was measured (1 pt.) Tested for potential non-linear relationships between green space and outcome, for instance, splitting green space into deciles or tertiles (1 pt.) Corrected for correlation between variables using a reasonable cut-off value (VIF < 3.0) (1 pt.) Did not consider pairwise error rates when reporting a large number of analyses, which affect Type I (false positive) error rates [41] (−1 pt.) For geospatial analyses, did NOT control for spatial autocorrelation, which results in correlated residuals and unreliable model results [42] (−1 pt.) For multi-year studies, did NOT control for temporal autocorrelation, which also lead to correlated residuals and unreliable model results [42] (−1 pt.)
Exposure assessment (for geospatial studies that rely on large datasets to measure green space)	Multiple seasons of green space data to control for seasonal fluctuations in measurement [43] (1 pt.) Multiple years of green space data to control for annual fluctuations in climate affecting measurement [44,45] (1 pt.) High resolution green space data (more than 50 m = 0 pt., 20 m to 50 m = 1 pt., 1 m or less, 2 pt.) to limit under- or over-estimating green space quantity across urban-rural gradients [46] Green space data not aligned in time with educational outcomes, for example green space data from 2004 and educational outcomes from 2012 [47] (−1 pt.)

**Table 2:** Description of study sample, outcome, and green space measure stratified by research design.

Citation	Sample Size	Geographic Context	Grade Level and Age	Green Space Measure(s)	Academic Outcome
Observational (*n* = 12)					
Beere & Kingham (2017) [47]	838 public schools	Cities in New Zealand	1–6th (6–12 years old 1)	Tree cover from a local land cover database (resolution not reported) from one year on school parcel and in attendance zone	Math, reading, and writing standardized test scores
Browning et al., 2018 [58]	404 public schools	Chicago, Illinois, United States	3rd (8 or 9 years old)	NDVI-derived greenness from MODIS (250 m resolution) over six years in spring, summer and fall (March, July, October) at 250, 500, 1000, and 3000 m radial buffers	Math and reading standardized test scores
Hodson & Sander (2017) [55]	222 public schools	Twin Cities, Minnesota, United States	3rd (8 or 9 years old)	Grass, shrub, and tree cover from NLCD (30 m resolution) in one year on school parcel and in attendance zone	Math and reading standardized test scores
Kuo et al., 2018 [28]	318 public schools	Chicago, Illinois, United States	3rd (8 or 9 years old)	Grass/shrub and tree cover from UTC (0.6 m resolution) in one year on school parcel and in attendance zone	Math and reading standardized test scores
Kweon et al., 2017 [54]	219 public schools	Washington, D.C., United States	2–10th (7 to 16 years old)	Grass/shrub and tree cover from UTC (0.6 m resolution) in one year on school parcel	Math and reading standardized test scores
Leung et al., 2019 [59]	3054 public schools	Massachusetts, United States	3–10th (ages 8–16)	NDVI-derived greenness from MODIS (250 m resolution) over eight years in spring and fall at 250, 500, 1000, and 2000 m radial buffers; Green land cover from a local database (0.5 m resolution) in one year at 250, 500, 1000, 2000 m radial buffers	Math and reading standardized test scores
Li et al., 2019 [60]	624 public schools	Illinois, United States	9–12th (ages 14–18)	Tree canopy cover from NLCD (30 m resolution) in one year at 400, 800, 1600, 3200, and 4800 m radial buffers	American College Test (ACT), a standardized test administered at the end of high school to evaluate preparation for college, which includes math, reading, and science; End-of-semester grades as determined by percent of students on-track for college with no more than one “F” letter grade after at least ten semesters of high school
Markevych et al., 2018 [51]	2429 students	Munich and Wesel areas, Germany	NR (age 10 and age 15)	NDVI-derived greenness from MODIS (250 m resolution) over eight years in summer months (May to August) at 500 and 1000 m radial buffers; Tree cover from Copernicus (20 m resolution) [62] at 500 and 1000 m radial buffers; Green land cover from local land use dataset for one year	Math and reading standardized test scores
Matsuoka, 2010 [48]	101 public schools	Southeast Michigan, United States	9–12th (ages 14–18)	Green view from cafeteria window; Grass cover on school parcel from aerial imagery	Michigan college preparatory exam for high school students; End-of-semester grades as determined by graduation rates, which require minimum letter grade average [63]
Sivajarah et al., 2018 [61]	387 public schools	Toronto, Ontario, Canada	3th and 6th (ages 8–9 and 11–12)	Tree canopy cover from UTC (0.6 m resolution) in one year on the school parcel; Number tree species and biodiversity from tree inventory	Math, reading, and writing standardized test scores
Tallis et al., 2018 [53]	495 public schools	California, United States	5th (ages 10–11)	NDVI-derived greenness and agricultural cover from NAIP (1 m resolution) in one year in summer at 50, 100, 300, 500, 750, and 1000 m radial buffers	Composite index of math, reading, and writing standardized test scores
Wu et al., 2014 [52]	6333 public schools	Massachusetts, United States	3rd (8 or 9 years old)	NDVI-derived greenness from MODIS (250 m resolution) over six years in spring, summer and fall (March, July, October) at 250, 500, 1000, and 3000 m radial buffers	Math and reading standardized test scores
**Experimental (*n* = 1)**					
Benfield et al. (2015) [49]	567 students	University in Pennsylvania, United States	College (age M = 18.9, SD = 1.57)	Green view vs. fogged view (no view but daylight present) from classroom windows	End-of-semester grades

^1^ ages reported are those typically associated with these grade levels in the United States [62], NR = not reported, MODIS = [63], NAIP = U.S. Department of Agriculture National Agriculture Imagery Program [64], UTC = Urban Tree Canopy Assessment [65,66], NLCD = National Land Cover Database [67], Note: For more details on green space measures, see [68,69].

## Appendix A

**Table A1:** Search expressions by database used in the review.

Database	Keyword Search
Web of Science	ALL FIELDS: ((“green space” OR “greenness” OR “greenspace” OR “tree cover*” OR “natural environment*” OR “nearby nature”) AND (“academic performance” OR “academic achievement” OR “test score*” OR “standardized test*” OR “semester grade*”)). Indexes: SCI-EXPANDED, SSCI, A&HCI, CPCI-S, CPCI-SSH, BKCI-S, BKCI-SSH, ESCI, CCR-EXPANDED, IC. ^a^
Scopus	TITLE-ABS-KEY ((“green space” OR “greenness” OR “greenspace” OR “tree cover*” OR “natural environment*” OR “nearby nature”) AND (“academic performance” OR “academic achievement” OR “test score*” OR “standardized test*” OR “semester grade*”)) AND DOCTYPE (ar)
Education Resources Information Center (ERIC)	(“green space” OR “greenness” OR “greenspace” OR “tree cover*” OR “natural environment*” OR “nearby nature”) AND (“academic performance” OR “academic achievement” OR “test score*” OR “standardized test*” OR “semester grade*”)

^a^ These constitute the Web of Science Core Collection.

**Table A2:** Outcomes and green space measures identified in the selected articles.

First Author	Academic Outcome	Green Space Measure	Distance	Association between Green Space and Outcome
Beere	Math	Green land	Schoolyard	Negative
Beere	Math	Green land	3000 m	Negative
Beere	Reading	Green land	Schoolyard	Negative
Beere	Reading	Green land	3000 m	Negative
Beere	Writing	Green land	Schoolyard	Negative
Beere	Writing	Green land	3000 m	Negative
Browning	Math	Greenness	500 m	Negative
Browning	Math	Greenness	3000 m	Negative
Browning	Math	Greenness	250 m	Negative
Browning	Math	Greenness	1000 m	Negative
Hodson	Math	Grass	3000 m	Null
Hodson	Math	Shrub	3000 m	Null
Hodson	Math	Tree	3000 m	Null
Hodson	Reading	Grass	3000 m	Null
Hodson	Reading	Shrub	3000 m	Null
Hodson	Reading	Tree	3000 m	Positive
Kuo	Math	Tree	Schoolyard	Positive
Kuo	Math	Tree	3000 m	Null
Kuo	Reading	Tree	Schoolyard	Null
Kuo	Reading	Tree	3000 m	Null
Kweon	Math	Grass	Schoolyard	Null
Kweon	Math	Tree	Schoolyard	Positive
Kweon	Reading	Grass	Schoolyard	Null
Kweon	Reading	Tree	Schoolyard	Positive
Leung	Math	Green land	500 m	Positive
Leung	Math	Green land	250 m	Positive
Leung	Math	Green land	2000 m	Positive
Leung	Math	Green land	1000 m	Positive
Leung	Math	Greenness	500 m	Positive
Leung	Math	Greenness	250 m	Positive
Leung	Math	Greenness	2000 m	Positive
Leung	Math	Greenness	1000 m	Positive
Leung	Reading	Green land	500 m	Positive
Leung	Reading	Green land	250 m	Null
Leung	Reading	Green land	2000 m	Positive
Leung	Reading	Green land	1000 m	Positive
Leung	Reading	Greenness	500 m	Positive
Leung	Reading	Greenness	250 m	Positive
Leung	Reading	Greenness	2000 m	Positive
Leung	Reading	Greenness	1000 m	Positive
Li	College	Tree	500 m	Positive
Li	College	Tree	3000 m	Positive
Li	College	Tree	3000 m	Positive
Li	College	Tree	250 m	Positive
Li	College	Tree	2000 m	Positive
Li	College	Tree	2000 m	Positive
Li	Grades	Tree	500 m	Null
Li	Grades	Tree	3000 m	Null
Li	Grades	Tree	3000 m	Positive
Li	Grades	Tree	250 m	Positive
Li	Grades	Tree	2000 m	Null
Li	Grades	Tree	2000 m	Null
Markevych	Math	Agriculture	500 m	Null
Markevych	Math	Agriculture	1000 m	Null
Markevych	Math	Green land	500 m	Null
Markevych	Math	Green land	1000 m	Null
Markevych	Math	Greenness	500 m	Null
Markevych	Math	Greenness	500 m	Null
Markevych	Math	Greenness	1000 m	Null
Markevych	Math	Greenness	1000 m	Null
Markevych	Math	Tree	500 m	Null
Markevych	Math	Tree	500 m	Null
Markevych	Math	Tree	500 m	Null
Markevych	Math	Tree	1000 m	Null
Markevych	Math	Tree	1000 m	Null
Markevych	Math	Tree	1000 m	Null
Markevych	Reading	Agriculture	500 m	Null
Markevych	Reading	Agriculture	1000 m	Null
Markevych	Reading	Green land	500 m	Null
Markevych	Reading	Green land	1000 m	Null
Markevych	Reading	Greenness	500 m	Null
Markevych	Reading	Greenness	500 m	Null
Markevych	Reading	Greenness	1000 m	Null
Markevych	Reading	Greenness	1000 m	Null
Markevych	Reading	Tree	500 m	Null
Markevych	Reading	Tree	500 m	Null
Markevych	Reading	Tree	500 m	Null
Markevych	Reading	Tree	1000 m	Null
Markevych	Reading	Tree	1000 m	Null
Markevych	Reading	Tree	1000 m	Null
Matsuoka	College	Grass	Schoolyard	Null
Matsuoka	College	Green land	View	Positive
Matsuoka	Grades	Grass	Schoolyard	Null
Matsuoka	Grades	Green land	View	Positive
Sivarajah	Math	Tree	Schoolyard	Null
Sivarajah	Reading	Tree	Schoolyard	Null
Sivarajah	Writing	Tree	Schoolyard	Positive
Tallis	Math	Agriculture	Schoolyard	Null
Tallis	Math	Agriculture	500 m	Null
Tallis	Math	Agriculture	1000 m	Null
Tallis	Math	Greenness	Schoolyard	Null
Tallis	Math	Greenness	500 m	Null
Tallis	Math	Greenness	1000 m	Null
Tallis	Math	Tree	Schoolyard	Null
Tallis	Math	Tree	500 m	Null
Tallis	Math	Tree	1000 m	Null
Tallis	Reading	Agriculture	Schoolyard	Null
Tallis	Reading	Agriculture	500 m	Null
Tallis	Reading	Agriculture	1000 m	Null
Tallis	Reading	Greenness	Schoolyard	Null
Tallis	Reading	Greenness	500 m	Null
Tallis	Reading	Greenness	1000 m	Null
Tallis	Reading	Tree	Schoolyard	Null
Tallis	Reading	Tree	500 m	Null
Tallis	Reading	Tree	1000 m	Null
Tallis	Writing	Agriculture	Schoolyard	Null
Tallis	Writing	Agriculture	500 m	Null
Tallis	Writing	Agriculture	1000 m	Null
Tallis	Writing	Greenness	Schoolyard	Null
Tallis	Writing	Greenness	500 m	Null
Tallis	Writing	Greenness	1000 m	Null
Tallis	Writing	Tree	Schoolyard	Null
Tallis	Writing	Tree	500 m	Null
Tallis	Writing	Tree	1000 m	Null
Wu	Math	Greenness	500 m	Positive
Wu	Math	Greenness	250 m	Positive
Wu	Math	Greenness	2000 m	Null
Wu	Math	Greenness	1000 m	Positive
Wu	Reading	Greenness	500 m	Null
Wu	Reading	Greenness	250 m	Positive
Wu	Reading	Greenness	2000 m	Null
Wu	Reading	Greenness	1000 m	Null
Wu	Reading	Greenness	1000 m	Null
